# Computational Analysis of Reciprocal Association of Metabolism and Epigenetics in the Budding Yeast: A Genome-Scale Metabolic Model (GSMM) Approach

**DOI:** 10.1371/journal.pone.0111686

**Published:** 2014-11-03

**Authors:** Ali Salehzadeh-Yazdi, Yazdan Asgari, Ali Akbar Saboury, Ali Masoudi-Nejad

**Affiliations:** Laboratory of Systems Biology and Bioinformatics (LBB), Institute of Biochemistry and Biophysics, University of Tehran, Tehran, Iran; University of Erlangen-Nuremberg, Germany

## Abstract

Metaboloepigenetics is a newly coined term in biological sciences that investigates the crosstalk between epigenetic modifications and metabolism. The reciprocal relation between biochemical transformations and gene expression regulation has been experimentally demonstrated in cancers and metabolic syndromes. In this study, we explored the metabolism-histone modifications crosstalk by topological analysis and constraint-based modeling approaches in the budding yeast. We constructed nine models through the integration of gene expression data of four mutated histone tails into a genome-scale metabolic model of yeast. Accordingly, we defined the centrality indices of the lowly expressed enzymes in the undirected enzyme-centric network of yeast by CytoHubba plug-in in Cytoscape. To determine the global effects of histone modifications on the yeast metabolism, the growth rate and the range of possible flux values of reactions, we used constraint-based modeling approach. Centrality analysis shows that the lowly expressed enzymes could affect and control the yeast metabolic network. Besides, constraint-based modeling results are in a good agreement with the experimental findings, confirming that the mutations in histone tails lead to non-lethal alterations in the yeast, but have diverse effects on the growth rate and reveal the functional redundancy.

## Introduction

Biological systems contain many highly interconnected processes that function in a coordinated fashion to produce cellular behavior. Therefore, understanding the biological networks and their crosstalk properties is important to put an accurate interpretation on the complex nature of biological systems. Metabolism and epigenetics are two central biological processes that are vital to the organisms' survival and reproduction. Metabolism is a complex network of dynamical biochemical reactions empowering organisms to grow, reproduce and maintain their integrity [1]. Epigenetic mechanisms, as a constituent of gene expression regulation machinery, alter transcriptional activities of various genes, independently of changes in their nucleotides sequences [2]. These mechanisms include modifications of DNA and histone proteins, such as different combinations of histone acetylation, methylation and phosphorylation which shape, the so-called histone code' and DNA methylation. Ultimately, these modifications lead to transcriptionally active or inactive chromatin [3]. Growing evidence suggests the possible link between different metabolic states of living systems and the epigenetic modifications [4]. For instance, histone acetylation is under control of changes in the intracellular concentration of acetyl-CoA [5]. On the other hand, some of these epigenetic modifications change the metabolic gene expression patterns under varying conditions [6]. Furthermore, recent studies propose that the impairments in the interface between epigenetics and metabolism are strongly associated with development of cancers [7] and metabolic syndromes [8]. Consequently, the cellular metabolism and the epigenetic modifications are not independent entities, and they would better be viewed as an integrated discipline, metaboloepigenetics that focuses on the crosstalk between them [9]. To illustrate the genotype-phenotype relationship of metabolism, a complete map of biochemical reactions and their comprehensive connections in a cell is required [10]. The Genome-Scale Metabolic Model (GSMM) is a mathematical framework, to gain a comprehensive understanding of physiology and the metabolic capacities of the cell, as well as being used for integrative data analysis of genetic, epigenetic and metabolism in combination [11]. Following the introduction of GSMM, the integration of gene expression data into the GSMM, was the new challenge for a better prediction of the metabolic cell fate. The integrative data approach, leads to a deeper understanding of the occurrence of certain changes in different conditions and creates condition- and tissue-specific models [12], [13]. For the first time, Covert and Palsson [14] addressed this issue in 2002. In 2004, Akesson et al. [15] used gene expression data as an additional constraint on the metabolic fluxes in yeast. Afterward, different algorithms were developed for tackling this challenge; GIMME [16], E-Flux [17], Moxley [18], MADE [19], RELATCH [20], INIT [21] and mCADRE [22]. Recently, advantages and disadvantages of different approaches of integration of expression data into constraint-based modeling have been evaluated [23]. Due to the availability of curated data [24], we focused on *Saccharomyces cerevisiae*, the budding yeast, as a model for investigating the global influences of epigenetic modifications on metabolism. The first budding yeast GSMM, iFF708, [25] was released in 2003. This model consists of 708 genes and 1175 reactions. Afterward, the improved versions of yeast GSMM were reconstructed; iND750 [26], iLL672 [27], iLN800 [28], iMM904 [29], improved iMM904 [30], and yeast 5 [31] released in the standard format using jamboree approach [32] which is the most up to date version.

In this study, we used the GSMM of *S. cerevisia*e, *Yeast_6.01,* as a scaffold, consisting of 889 genes, 1889 reactions (150 reactions are irreversible), 1456 metabolites and the standard biomass equation. Then we analyzed the nine gene expression profiles of four different states of mutated histones tails (H2A, H2B, H3, and H4) extracted from Gene Expression Omnibus (GEO) [33]. Subsequently, we constructed nine models, by integrating fold change values of the significantly lowly expressed reactions of each gene expression profile, as an additional constraint on metabolic fluxes. Afterward, we have tried to explore possible relation between topological analysis of metabolic network and down-regulated genes with this assumption that down-regulated genes could affect and control the yeast metabolic network. Then, Flux Balance Analysis (FBA), as a constraint-based modeling approach has been used to compute and compare [34], [35] the impact of the mutated histone tails on every reaction flux, the global metabolic fluctuations and the growth rate. The results verifies the prior experimental findings, showing that the histone tails are not essential for the viability of yeast but have a large impact on a vast range of metabolic reactions, which reveals the functional redundancy of the histone tails and their ability to regulate their own metabolite sources [36].

## Materials and Methods

### 2.1. The GSMM of yeast and gene expression data

In this study, we used microarray gene expression profiles and RNA-Seq data of mutated histone tails [37]–[40] of *S. cerevisiae* extracted from GEO database. The GSE accession numbers and their categorizations are listed below. The GSMM of yeast in SBML format (Systems Biology Markup Language), a representative format for mathematical models of biological processes such as metabolic network, was obtained from http://www.comp-sys-bio.org/yeastnet. The metabolic states of yeast have been studied by integration of gene expression data into the *Yeast_6.01* GSMM as a scaffold model.

**GSE1639:** Consists of gene expression profiles of H3 and H4 mutated tail. [37].

Group 1: H3 deleted tail.

Group 2: H3 substituted tail, lysine substituted with glutamine residues in histone 3 tail.

Group 3: H4 substituted tail, lysine substituted with glutamine residues in histone 4 tail.

Group 4: H4 deleted tail.

**GSE3806:** Consists of gene expression profiles of H2B mutated tail. [38].

Group 5: H2B deleted tail.

Group 6: H2B substituted tail, lysine substituted with glutamine residues in histone 2B tail.

**GSE7337:** Consists of gene expression profile of H2A deleted tail. [39].

Group 7: H2A deleted tail.

**GSE7338:** Consists of gene expression profile of H2A substituted tail. [39].

Group 8: H2A substituted tail, lysine substituted with glutamine residues in histone 2A tail.

**GSE29293:** Consists of gene expression profile of H3 depletion. [40].

Group 9: H3 depletion.

### 2.2. Gene expression analysis

Microarray gene expression data analysis for each given group has been done by GeWorkbench 2.4.0 software [41]. RNA-Seq data analysis has been done according to methodology explained in [42]. Statistical significance of up- and down-regulated genes computed by t-test for the Wild Type (WT) and its corresponding mutated histone tail on log_2_-normalized data (using WT and mutated histone tail data sets as case and control, respectively). The differential expression of a gene defined significant, if *p-value* <0.01 and the negative fold change value was indicative of the down-regulated genes. The SBML file of *Yeast_6.01* was converted into COBRA (Constraints Based Reconstruction and Analysis) model structure by COBRA toolbox for subsequent analysis and the lowly expressed reactions determined according to gene-protein-reaction relationship (GPR) in the COBRA model of *Yeast_6.01*.

### 2.3. Model construction

FBA, a constraint-based modeling approach, calculates the flow of metabolites through a metabolic network. This method allows predicting the rate of production of a metabolite or the growth rate of an organism. FBA includes delineating constraints on the network, based on environmental, physicochemical, regulatory, enzyme capacity and thermodynamics principles for shrinking the solution space. Integration of transcriptomic data into GSMM is a way to generate better predictive computational models through adding an extra biologically meaningful constraint and limiting the solution space of the GSMM. Blazier and Papin in a comprehensive review, summarized the differences, limitations and advantages of all integration algorithms [43]. Considering biological concepts, there are some limitations in the different integration algorithms. For example, the GIMME algorithm reduces gene expression data into binary states (0 and 1 for on and off state of an enzyme, respectively) and the iMAT algorithm [44] into three states (−1, 0, and 1 for lowly expressed, moderately expressed, and highly expressed enzymes, respectively). It is not biologically acceptable that the down-regulated genes and their corresponding fluxes removed from the model, because lowly expressed is not equivalent with the gene silencing. In MADE approach [19], there is no exact threshold to determine which reaction is highly expressed and which one is lowly expressed and in E-Flux [17] there is no function to convert gene expression level into fluxes.

**Figure 1:**
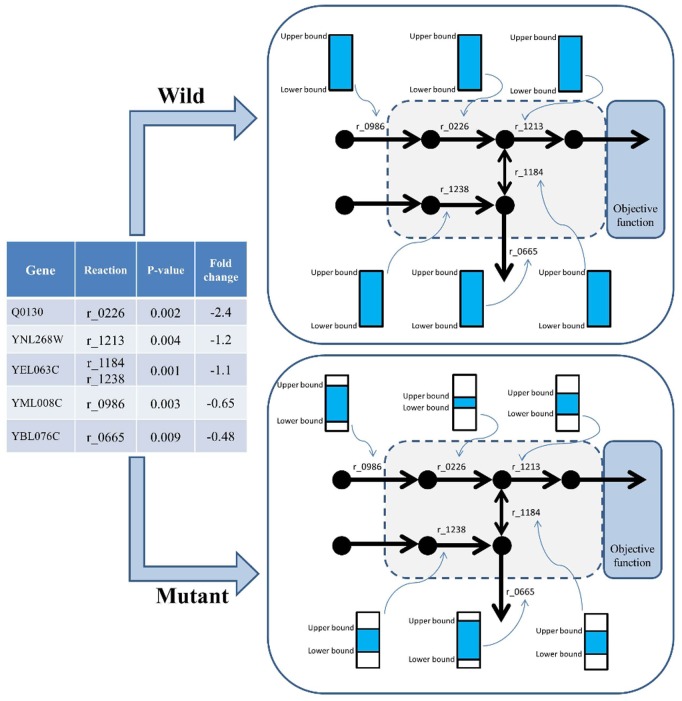
This figure is a schematic workflow of our method for GSMM construction. The significant down-regulated gene (*p-value* <0.01) and the corresponding reactions (according to GPR) were determined. (In this model, 5 genes and 6 reactions were identified). Afterward, we have restricted the fluxes of the given reactions according to their fold change values. For example if the fold change value is −2, we have restricted the upper bound and lower bound of the corresponding flux to one quarter of the corresponding WT.

Totally, there are two main classes for creating condition-specific models; switch- and valve-based approaches. In a switch-based approach, the lowly expressed genes removed from constrained model by adjusting their corresponding reactions boundaries (lower and upper bounds) to zero, while in a valve-based approach, the activity of lowly expressed genes decrease by reducing the corresponding reaction boundaries according to expression values [45]. In our integration method, we followed the valve-based approach. First, we used the simulation results of the unconstrained initial model (*Yeast_6.01*) to identify the reaction’s fluxes (which matches fluxes observed in vivo) and established the boundaries of the WT model according to the reaction fluxes of the initial model [31]. Then, we imposed the gene expression data of nine groups on these fluxes to construct new models. It means that, the solution space of the constructed GSMMs has been shrunk by adjusting the reaction’s fluxes of initial model according to their fold change values of lowly expressed genes. In other words, the upper and lower bounds of our new constructed models are the reduced simulated reaction’s fluxes of initial model. However, to represent up-regulated genes, we have to increase the flux ranges of the corresponding reactions which will not have any impact on the FBA-based solution. Therefor we excluded the up-regulated genes from our study. We have used *p-value* as a threshold for identification of highly and lowly expressed genes. Finally, for not reducing the gene expression data into binary or three states, we used fold change values as a quantitative parameter to constraint the fluxes. Figure 1 shows that how expression data integrated into GSMM.

Moreover, we used the standard biomass equation as an objective function of the yeast growth rate. To investigate the comprehensive metabolic properties of the models, several COBRA utilities have used. All GSMMs are available in the File S2.

### 2.4. Topological analysis

Centrality analysis has been carried out on the undirected enzyme-centric network [46] of *Yeast_601* using CytoHubba plug-in in Cytoscape [47]. We have used twelve centrality indices: Maximal Clique Centrality (MCC), Density of Maximum Neighborhood Component (DMNC), Maximum Neighborhood Component (MNC), Degree, Edge Percolated Component (EPC), Bottleneck, Eccentricity, Closeness, Radiability, Betweenness, Stress and Clustering Coefficient. Then, the lowly expressed enzymes of nine constructed GSMMs, were sorted based on their centrality indices. (For more information, see part A in the File S1).

### 2.5. COBRA utilities that have been used in this study

Among various software for calculating the FBA, we used the well-known MATLAB toolbox, COBRA, and a standard objective function (maximization of biomass equation) for evaluating the metabolic models. After running the *optimizeCbModel* function in the COBRA toolbox, four main output structures are built: *f*, *x*, *y*, and *w*, where *f* is optimal objective value, *x* is containing reaction fluxes of each reaction in the model, *y* is a vector of shadow prices, and *w* is a vector of reduced costs.

Then, we calculated the number of carrying-flux reactions (negative and positive carrying-flux reactions) according to the *x* file of each model extracted after performing FBA.

Flux Variability Analysis (FVA) was carried out on all GSMMs to identify and compare the range of possible flux values (flux capacity) resulting in the same optimal objective value (maximum and minimum possible fluxes through a particular reaction). We compared the flux capacity of each reaction of a mutated histone tail models with the corresponding reaction of WT model. So, positive values indicate increased flux capacity in mutated histone tail models in comparison to corresponding WT model, whereas negative values indicate decreased flux capacity in mutated histone tail models in comparison to corresponding WT model. Finally, the increased and decreased ranges of flux values categorized in the yeast metabolic subsystems.

Single Gene Deletion used to compute the essential genes, which are important for the growth of the each model.

Parsimonious FBA (pFBA) used to categorize the metabolic reactions to the six groups according to their importance in FBA.

The *SingleGeneDeletion* function is implemented by constraining the flux of deleted gene to zero and then the flux distribution and maximal growth for the new phenotype simply calculated by FBA. If the maximal growth of the new phenotype is reduced, the gene will be essential. The pFBA method is a modified of FBA in which an extra constraint is added. In this approach, after maximizing the growth rate, the net metabolic flux through all gene-associated reactions will be minimized [35], [48]. According to the additional constraint (minimization of metabolic adjustment) in pFBA method, the number of non-essential metabolic genes decreases compared with single gene deletion method [48].

Then, we calculated the percentage of the lowly expressed enzymes of the nine constructed GSMMs in each category of pFBA and Single Gene Deletion. (For more information, see part B in the File S1).

**Figure 2:**
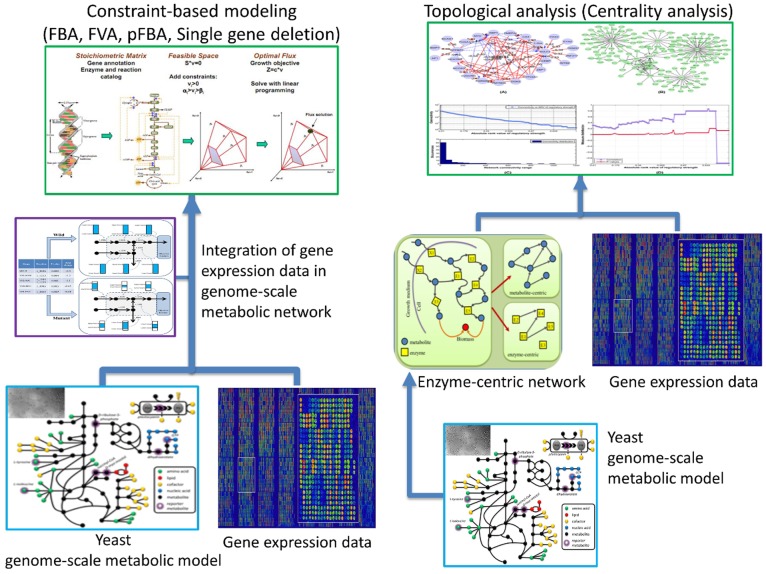
Shows the workflow of our study. This study consists of two parts; topological analysis and constraint-based modeling. In the topological analysis section, we used the twelve centrality indices of the undirected enzyme-centric network of yeast and examined the distribution of the down-regulated genes in the nine mutated histone tails profiles, extracted from geWorkbench software. In the constraint-based modeling section, we integrated gene expression of nine groups of the mutated histone tails profiles to the yeast GSMM and constructed new models. FBA, FVA, pFBA and Single gene deletion analyzed by COBRA toolbox.

Production of cofactors and biomass precursors: We used this capability of FBA for calculating the maximum yield of acetyl-CoA in GSMMs. Acetyl-CoA in nucleocytosolic compartment is the main donor of acetyl group in histone acetylation and is also used for de novo synthesis of fatty acid. In fact, histone acetylation and synthesis of fatty acids compete for the same acetyl-CoA pool. Acetyl-CoA synthetase is responsible for acetyl-CoA production and acetyl-CoA carboxylase, is an enzyme which carboxylates acetyl-CoA to form malonyl-CoA in de novo synthesis of fatty acids. Actually, acetyl-CoA carboxylase regulates the activity of acetyl-CoA synthetase [49]. For maximizing the production and regulation of acetyl-CoA, we set the objective function of the constructed models to acetyl-CoA carboxylase and acetyl-CoA synthetase reactions respectively, and turning the lower bound of these two reactions to 0 because these two reactions are known to be irreversible. Figure 2 illustrates the workflow of our study.

## Results

### 3.1. Gene expression results

In this study, we just considered the significantly up- and down- regulated metabolic genes extracted from GeWorkbench 2.4.0 software. Table 1 summarizes the basic information about up- and down-regulated metabolic genes. The table shows that the highest number of the significant metabolic genes was found in H3 deletion group, whereas the group 8, H2A substituted tail, had the lowest number of the significant metabolic genes. For constraint-based modeling, we took into account the down-regulated metabolic genes for further analysis. Afterward, the nine mutated histone tail models were constructed. All data are available in the File S3.

**Table 1:** Up and down significant metabolic genes in each group.

GEO accession	(Histone Modifications)	Total no. of GSMMSignificant genes	No. of Up-RegulatedGenes	No. of Down-Regulated genes	Annotation files
**1.GSE1639**	WT-H3 (Deletion1–28)	36	31	5	YG_S98.na32.annot
**2.GSE1639**	WT-H3(K4,9,14,18,23,27Q)	96	58	38	YG_S98.na32.annot
**3.GSE1639**	WT-H4 (K5,8,12,16Q)	35	18	17	YG_S98.na32.annot
**4.GSE1639**	WT-H4 (Deletion 2–26)	41	14	17	YG_S98.na32.annot
**5.GSE3806**	WT-H2B (Deletion 3–32)	8	3	5	YG_S98.na32.annot
**6.GSE3806**	WT-H2B (K-G)	12	3	9	YG_S98.na32.annot
**7.GSE7337**	WT-H2A (Deletion 4–20)	57	33	24	YG_S98.na32.annot
**8.GSE7338**	WT-H2A (K4,7G)	9	7	2	YG_S98.na32.annot
**9.GSE29293**	WT-H3 Depletion	135	130	5	

### 3.2. Topological analysis

**Figure 3:**
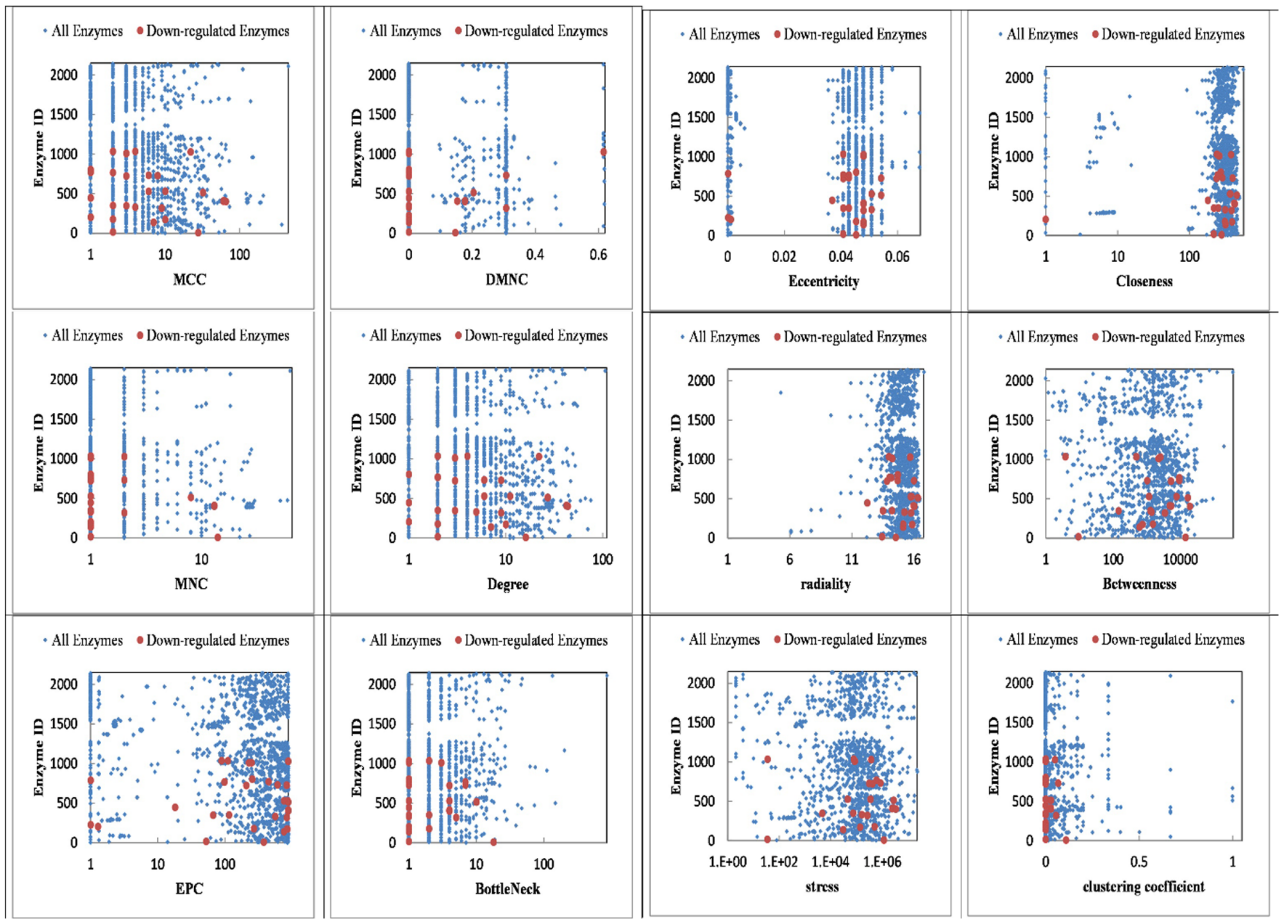
Shows the distribution of down-regulated enzymes in enzyme-centric network of yeast in H4 substituted tail model. The x- and y-axis indicate the centrality score and the enzyme ID of the H4 substituted tail model, respectively.

Centrality indices are a global property of a network that ranks the graph nodes according to their importance in the network. The higher the rank the more important the node is in the network, indicating that it may play key roles in controlling cellular behavior [50]. Twelve centrality indices of the undirected enzyme-centric network of yeast were computed by cytoHubba. Results show that the lowly expressed enzymes are in the top rank of centrality indices of each given model. The File S4, lists the lowly expressed enzymes among the 100 top ranked of twelve different centrality indices. Figure 3 shows the distribution of the lowly expressed enzymes in undirected enzyme-centric network of yeast in the H4 substituted tail model as an example. Results show that the properties of yeast metabolic network could change despite its scale-freeness.

### 3.3. Constraint-based modeling results

#### 3.3.1. FBA and comparison of all reaction fluxes in the nine models

**Figure 4:**
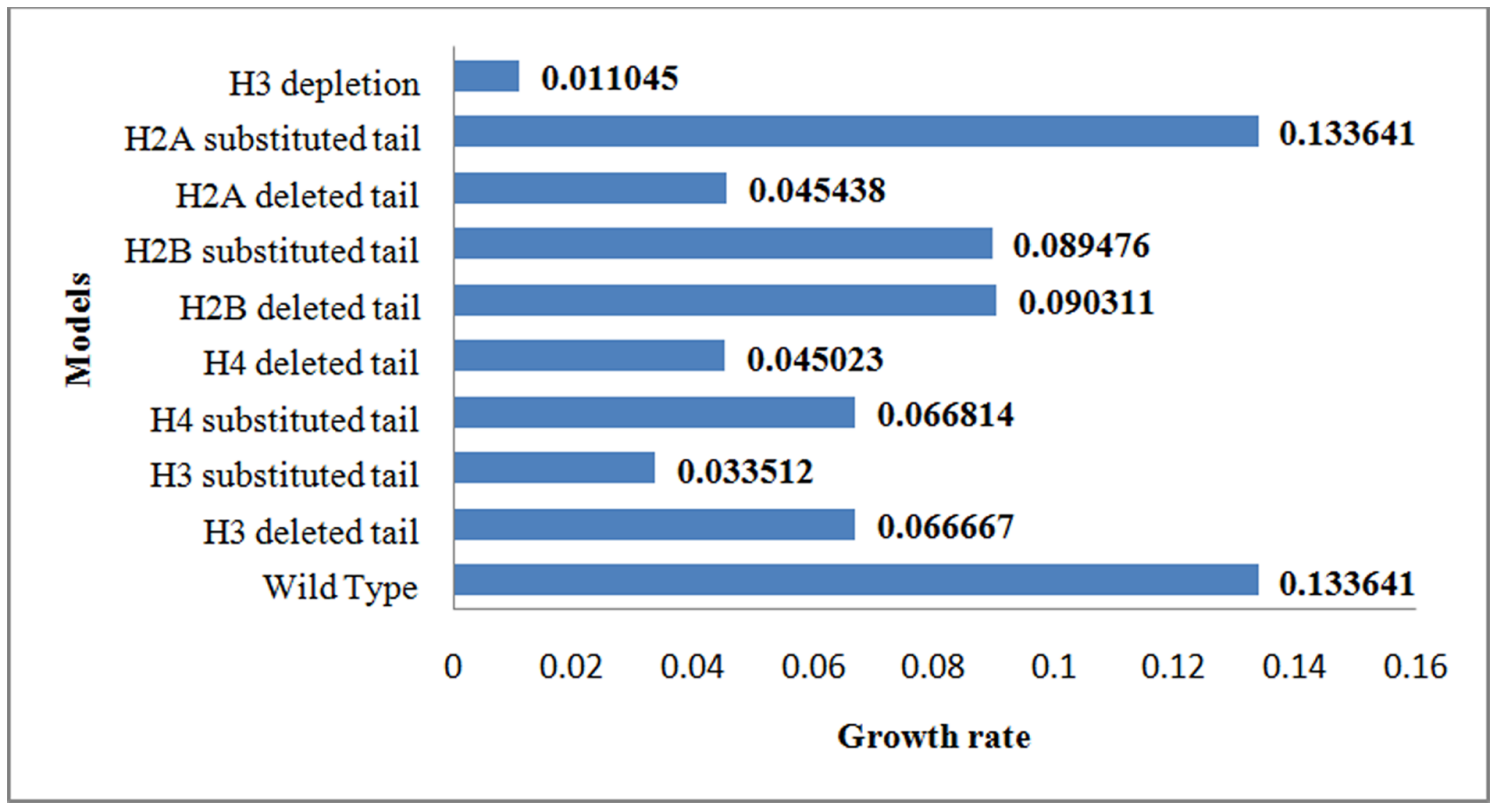
Shows the growth rate (the optimal objective value) of all constructed GSMMs calculated by FBA. The unit of the growth rate is mmol gDW^−1^ hr^−1^ (milimoles per gram dry cell weight per hour).

After model construction, to scrutinize the impact of mutated histone tails on the cell growth rate, FBA was carried out for all nine constructed GSMMs while the objective function was a standard biomass function. The biomass function is a hypothetical reaction that experimentally determines and quantifies the specific growth rate of the cell. This reaction reflects the needs of the cell in order to make 1 gr of cellular dry weight. Figure 4 summarizes the optimal objective values of all constructed models. Results show that the different mutated histone tails have diverse effects on the growth rate of the corresponding models. The H3 depletion model, has the lowest value while the H2A substituted tail model, has no changes in the optimal objective value.

**Figure 5:**
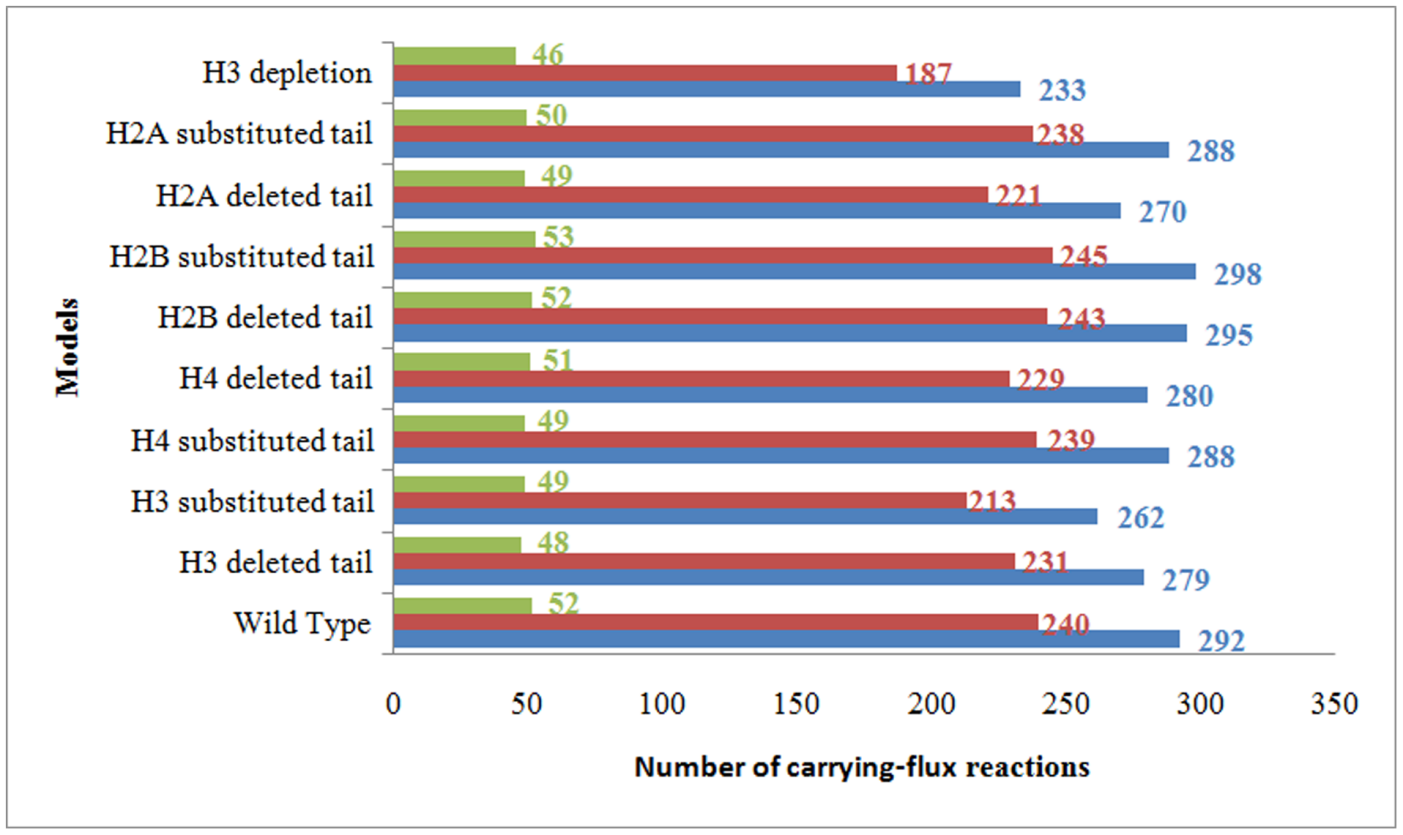
Comparison of the number of all, positive and negative carrying-flux reactions (blue, brown and green bars, respectively) in all constructed GSMMs.

All the computed optimal reaction fluxes of the nine models were compared with the WT model (as it has been described in the method section 2.5). Results as indicated in Figure 5, show that the number of carrying-flux reactions of all models has been decreased in comparison with the WT model, except in the H2B deleted and substituted tail models. The numbers of the negative carrying-flux reactions (according to the direction of reversible flux) have almost no change in comparison with the positive carrying-flux reactions. It means that in the H2B model, despite the increase in the carrying-flux numbers, the optimal objective value decreases. In other words, H2B modifications have a direct effect on the growth rate.

#### 3.3.2. FVA

Biological systems often contain redundancies that contribute to their robustness. FVA is a valuable method to examine the redundancy of metabolic network by calculating the full range of the numerical values (maximum and minimum possible fluxes) for each reaction. FVA was carried out for each model and subsequently compared to the WT model. Then, increased and decreased flux range of each reaction within the metabolic subsystems of yeast were determined. Table 2 summarizes the increased flux range of the metabolic subsystems of each model. As results in Table 3 show, the main effects of histone tail modifications are on the increasing of flux range.

**Table 2:** Increased flux range of yeast metablic subnetwork in the 9 mutated histone tail models.

Metabolic subsystem	Models
**2-ketoglutarate dehydrogenase complex**	1- 3- 4- 5- 9
**acetoin biosynthesis**	1- 3- 4- 5- 6- 9
**arginine biosynthesis**	1- 2- 4- 6
**arginine degradation**	1- 3- 4- 5- 6- 9
**beta-alanine biosynthesis**	1- 2
**butanediol biosynthesis**	1- 2- 3- 4- 5- 6- 9
**chorismate biosynthesis**	1- 2- 3- 4- 5- 9
**de novo biosynthesis of purine nucleotides**	1- 2- 3- 5- 9
**de novo biosynthesis of pyrimidine ribonucleotides**	1- 2- 3- 6- 9
**ergosterol biosynthesis**	1- 5- 6- 9
**fatty acid biosynthesis**	1- 2- 3- 4- 5- 6- 9
**folate biosynthesis**	1- 2- 3- 4- 5- 6- 9
**formaldehyde oxidation**	1- 2- 3- 4- 6- 9
**glutamate biosynthesis**	1- 2- 3- 4- 5- 6- 9
**glutamate degradation**	1- 3- 4- 5- 6- 9
**glutathione-glutaredoxin redox reactions**	1- 3- 4- 5- 6- 9
**glycine biosynthesis**	1- 2- 3- 4- 5- 6- 9
**glycolysis/gluconeogenesis**	1- 2- 3- 4- 5- 6- 9
**glyoxylate cycle**	1- 2- 3- 4- 5- 6- 9
**hexaprenyl diphosphate biosynthesis**	1- 5- 6- 9
**histidine biosynthesis**	1- 2- 3- 5- 9
**homoserine biosynthesis**	1- 3- 4- 5- 6- 9
**isoleucine biosynthesis**	1- 2- 3- 4- 5- 6- 9
**leucine biosynthesis**	1- 2- 3- 4- 5- 6- 9
**lysine biosynthesis**	1- 2- 3- 4- 5- 6- 9
**methionine salvage pathway**	1- 3- 4- 5- 9
**mevalonate pathway**	1- 2- 3- 4- 5- 6- 9
**nonoxidative branch of the pentose phosphate pathway**	1- 2- 3- 4- 5- 6- 9
**oxidative branch of the pentose phosphate pathway**	1- 2- 3- 4- 5- 6- 9
**p-aminobenzoate biosynthesis**	1- 2- 3- 5- 9
**pantothenate and coenzyme A biosynthesis**	1- 3- 4- 5- 6- 9
**periplasmic NAD degradation**	1- 2- 3- 9
**phenylalanine biosynthesis**	1- 2- 3- 4- 5- 6- 9
**phosphatidate biosynthesis**	2- 4- 9
**proline biosynthesis**	1- 2- 3- 4- 5- 6- 9
**putrescine biosynthesis**	1- 3- 4- 5- 9
**pyruvate dehydrogenase**	1- 2- 3- 4- 5- 6- 9
**S-adenosylmethionine biosynthesis**	1- 2- 3- 4- 5- 6- 9
**salvage pathways of purines and their nucleosides**	1- 2- 3- 9
**spermidine and methylthioadenosine biosynthesis**	1- 3- 4- 5- 9
**sulfate assimilation pathway**	1- 2- 3- 4- 5- 6- 9
**superpathway of glucose fermentation**	1- 3- 4- 5- 6- 9
**superpathway of histidine, purine, and pyrimidine biosynthesis**	1- 2- 3- 5- 9
**TCA cycle, aerobic respiration**	1- 3- 4- 5- 9
**thioredoxin system**	1- 2- 3- 4- 5- 6- 9
**tryptophan biosynthesis**	1- 2- 3- 5- 6- 9
**tryptophan degradation**	1- 2- 3- 4- 5- 6- 9
**tyrosine biosynthesis**	1- 2- 3- 4- 5- 6- 9
**tyrosine degradation**	1- 2- 3- 5- 6
**valine biosynthesis**	1- 3- 4- 5- 6- 9
**valine degradation**	1- 2- 3- 4- 5- 6- 9

Models 1–9 refer to the H3 deleted tail model, the H3 substituted tail model, the H4 substituted tail model, the H4 deleted tail model, the H2B deleted tail model, the H2B substituted tail model, the H2A deleted tail model, the H2A substituted tail model and the H3 depletion model, respectively.

**Table 3:** Decreased flux range of yeast metablic subnetwork in the 9 mutated histone tail models.

Metabolic subsystem	Models
**ATPase, cytosolic**	3–9
**de novo biosynthesis of purine nucleotides**	2- 3- 4- 6- 7
**de novo biosynthesis of pyrimidine deoxyribonucleotides**	4–5
**de novo NAD biosynthesis**	2–4
**fatty acid oxidation pathway**	2–6
**folate transformations**	2
**glycerol degradation**	2–6
**glycine cleavage complex**	2
**histidine biosynthesis**	4
**NADH dehydrogenase**	3
**periplasmic NAD degradation**	7
**phosphatidylinositol phosphate biosynthesis**	4
**salvage pathways of adenine, hypoxanthine and their nucleosides**	2- 3- 4
**salvage pathways of guanine, xanthine and their nucleosides**	4
**salvage pathways of purines and their nucleosides**	4
**salvage pathways of pyrimidine ribonucleotides**	2- 3- 4
**serine biosynthesis**	7
**TCA cycle, aerobic respiration**	2–6

Models 1–9 refer to the H3 deleted tail model, the H3 substituted tail model, the H4 substituted tail model, the H4 deleted tail model, the H2B deleted tail model, the H2B substituted tail model, the H2A deleted tail model, the H2A substituted tail model and the H3 depletion model, respectively.

#### 3.3.3. pFBA and single gene deletion results

pFBA was used to label all metabolic genes based on their ability to contribute to the optimal growth rate predictions. As results in Table 4 show, the lowly expressed enzymes in each model are important regarding to *SingleGeneDeletion* function and pFBA analysis.

**Table 4:** The percentage of the lowly expressed enzymes of nine constructed models in each category of pFBA and Single Gene Deletion.

Reaction in (GPR)	Single gene deletion	essential genes	pFBA optima	ELE	MLE	pFBA no-flux	Blocked
H3 deleted tail	33.33%	33.33%	16.7%	0	50%	0	0
H3 substituted tail	4%	16.8%	11.8%	13.9%	15.9%	5%	36.6%
H4 substituted tail	6.6%	13.3%	26.7%	23.3%	6.7%	0	30%
H4 deleted tail	4.1%	12.5%	16.7%	16.7%	8.3%	0	45.8%
H2B deleted tail	0	20%	40%	0	0	0	40%
H2B substituted tail	0	5%	0	30%	5%	10%	50%
H2A deleted tail	6.3%	19%	4.8%	6.3%	30.1%	4.8%	35%
H2A substituted tail	0	0	50%	0	0	0	50%
H3 repletion	16.6%	66.8%	0	16.6%	16.6%	0	0

#### 3.3.4. Production and regulation of acetyl-CoA

Table 5, shows the different turnover values of acetyl-CoA according to two reactions, acetyl-CoA synthetase and acetyl-CoA carboxylase reactions, (that are responsible for production and regulation of acetyl-CoA, respectively) in the ten models. All the mutated histone tail models changed the turnover values of this metabolite. Results show that the histone tails play a key role in production and regulation of acetyl-CoA.

**Table 5:** We set the objective function of the constructed models to the given reactions (acetyl-CoA synthetase and acetyl-CoA carboxylase reactions) and turned the lower bound to zero for maximizing the production and regulation reaction of acetyl-CoA.

	Model 0	Model 1	Model 2	Model 3	Model 4	Model 5	Model 6	Model 7	Model 8	Model 9
**Regulation**	1.5652	1.5652	0.1318	1.3358	1.3726	1.3643	1.5652	0.5209	1.5652	1.5319
**Production**	150.8571	25.75	144.4722	150.8571	73.25	144.0963	149.236	0.4	19.8	150.8571

The producing reaction of acetyl-CoA is catalyzed by acetyl-CoA synthetase, which is in turn regulated by acetyl-CoA carboxylase, an enzyme which catalyzes acetyl CoA conversion to malonyl-CoA, the first and rate-limiting reaction in de novo synthesis of fatty acids. The optimal objective values for acetyl-CoA regulation and production calculated by FBA for all models. Models 1–9 refer to the H3 deleted tail model, the H3 substituted tail model, the H4 substituted tail model, the H4 deleted tail model, the H2B deleted tail model, the H2B substituted tail model, the H2A deleted tail model, the H2A substituted tail model and the H3 depletion model, respectively.

## Discussion

Cell metabolism is dependent on different factors. Among those, some are related to the external metabolites such as nutrient cultures, while some are internal regulatory factors [51]. It has been shown that metabolites can regulate the chromatin-modifying enzymes activities and dynamical property of the epigenome can modify the gene expression pattern of the cell. These evidences, providing a direct link between metabolism and epigenetics. Scientific approaches that deal with gene expression analysis, which related to the metabolism have several different purposes. While some studies are directly targeting the theoretical foundations, others trigger specific biological questions. In this study, we aim to answer two main theoretical questions:

- Do the modifications of histone tails (i.e., histone tail deletion or the substitution of amino acid lysine with glutamine in histone tail) affect the whole metabolism of yeast? Moreover, if so, to what rate? In addition, whether the GSMMs are able to explain these changes?
- Is the nucleocytoplasmic acetyl-CoA, which is the main metabolite pool for acetylation of histones, under the control of histone tail modifications? Moreover, whether the epigenetic modifications can control the main source of acetylation?

To answer the first question, we used GSMM of yeast and nine gene expression profiles of the mutated histone tails. Determination of structural properties of a network and its nature is the first step to analyze the given network [52]. The oldest and the most complicated network in a living cell is metabolic network, which its topological characteristics are known. The metabolic network of yeast shows power-law degree distribution pointing to the fact that the network is robust to random failures and modifications in its structure [53], [54]. On the other hand, the essential genes are vital to maintenance cellular life and their deletion will result in lethality or infertility. Although, the main approach for separating these genes from non-essential genes is experiment, but there are some theoretical approaches for predicting these genes. Network biology, is one of the theoretical approaches, which determines the important nodes in a network by calculating the different centrality parameters. Analyzing the undirected enzyme-centric network of yeast according to twelve centrality indices, shows that some of the lowly expressed enzymes are very important for the network robustness, and suggesting that metabolism could be influenced by them. Concomitantly, pFBA and single gene deletion analysis, as two different theoretical approaches which especially applied to metabolic network, confirmed this expectation. The pFBA analysis categorizes all the reactions in the metabolic network to six clusters. The results of pFBA and single gene deletion show some of the lowly expressed enzymes are crucial for the yeast growth and can affect the metabolic network and metabolism. Therefore, concluded from these results that the connection between histone modifications and metabolism is not implausible. Pioneering works demonstrated that the histone tails or enzymes which are related to the acetylation of histone (e.g. histone acetyl transferase) control the metabolism of yeast, but are not essential for its viability [55]. For the quantification of this crosstalk behavior and gaining more information about how these modifications affect yeast metabolism, FBA has been done. The FBA results (when the objective function was the biomass equation) show considerable changes in the optimal flux values and the growth rate of the constructed models. Although these substantial changes differed in each model, but none of them is lethal. Unlike the growth rate of the H2A substituted tail model remained unchanged, the depletion of the H3 had the highest effect on the growth rate. Subsequently, the deleted and substituted H2B tail models showed the modest changes, while the substituted H3 and the H4 tail models showed more changes. This leads us to the conclusion that lysine in H3 and H4 as the target of histone modifications has a major role in the growth rate of yeast and the different H3 and H4 modifications can have different growth rate. The FBA results confirm the previous experimental results that the histone modifications were not lethal. In 2012 Kim et al., demonstrated that the N-terminal tails of histones reveal functional redundancy in the budding yeast [36]. The results calculated by FVA shows, that the most of the reaction flux has been changed in all nine models and display different metabolic patterns. The common increased range of flux in the subsystem of the mutated H3, H4 and H2B tail models indicated a functional redundancy in regulation of yeast metabolism including amino acid biosynthesis (e.g. glycine, histidine, homoserine, isoleucine, leucine, lysine, methionine, phenylalanine, proline, tryptophan, tyrosine and valine), as well as glycolysis, gluconeogenesis, glyoxylate cycle, pentose phosphate pathway and TCA cycle. By changing the objective functions of the models to a specific reaction, the maximum yields of important metabolites measured. In all models, our data indicates that acetyl-CoA turnover changes in dependence of mutated histone tails, which is potentially indicative of a change in its concentration. Indeed, it shows that the main source of acetylation is under control of epigenetic modifications. Finally, it is broadly accepted that the main sources of epigenetic changes are metabolites, which can be provided by different states of the cell metabolism. On the other hand, epigenetic modifications change the metabolic states of a cell. One of the essential reactions, underlying this change is acetyl-CoA synthetase. Therefore, histone tails have a feedback control on their main acetylation source, as a major target of epigenetic modifications. Thus, we can claim that histone tails are the key players of crosstalk between epigenetics and metabolism.

## Supporting Information

File S1 **A:** Centrality definitions used in this study. **B:** Definition of constraint-based analysis performed in the study.
(DOCX)

File S2 **Genome-Scale Metabolic Models (GSMMs) in MATLAB structure format.**
(RAR)

File S3 **Up- and down regulated genes and their corresponding reactions according to GPR in nine mutated histone tail models.**
(DOCX)

File S4 **The lowly expressed enzymes among the 100 top ranked centrality indices.**
(XLSX)
