# Supplementary material for: Computational Analysis of Reciprocal Association of Metabolism and Epigenetics in the Budding Yeast: A Genome-Scale Metabolic Model (GSMM) Approach
Source: PLoS One. 2014 Nov 3;9(11):e111686. doi: 10.1371/journal.pone.0111686 (PMC4218804; doi:10.1371/journal.pone.0111686)
Supplement: File S1 — A: Centrality definitions used in this study. B: Definition of constraint-based analysis performed in the study. (DOCX) [file pone.0111686.s001.docx]

**Part A**

**Topological Analysis: Centrality definitions**

Consider a network *G* = (*V*, *E*), where *V* and *E* are the vertex and the edge set, respectively. For a set *S*, its cardinalitydenotes by |*S*|. The length of a shortest path between vertices *u* and *v* is denoted as *dist*(*u*, *u*). Given a vertex *v*, the centralities of the vertex are shown as follows [1-4]:

| **Centrality** | **Mathematical Equation** | **Options** |
| --- | --- | --- |
| **MCC** | $\sum_{C\in S(v)} \left( \left\vert C \right\vert-1 \right)!$ | *S(v)*={*C*: a maximal clique which contains *v*} |
| **DMNC** | $\frac{\left\vert E\left( MNC(v) \right) \right\vert}{\left\vert V\left( MNC(v) \right) \right\vert^{\varepsilon}}$ | 1*<ε<*2 |
| **MNC** | $\left\vert MNC(v) \right\vert$ | *MNC(v)*=the maximum connected component of the $G\left[ N(v) \right]$ where *N(v)*=neighbors of a vertex *v* and $G\left[ N(v) \right]$ is an induced subgraph by *N(v)* |
| **Degree** | $deg(v)$ | $deg(v)$ denotes thenumber of the neighbours of vertex *v* |
| **EPC** | $\frac{1}{V}\sum_{t\in V} \left\langle\delta_{vt} \right\rangle$ | $\delta_{vt}=1$If vertices v and t are connected, and $\delta_{vt}=0$ otherwise. $\left\langle\delta_{vt} \right\rangle$ denotes the ensemble average of $\delta_{vt}$ |
| **BottleNeck** | $\sum_{s\in V} p_{s}(v)$ | Let $T_{s}$ be a shortest path tree rooted at s. $p_{s}\left( v \right)=1$ if more than $\left\vert V(T_{s}) \right\vert/4$ paths from s to other vertices in $T_{s}$ meet at the vertex *v*; otherwise $p_{s}\left( v \right)=0$ |
| **Eccentricity** | $\frac{p(v)}{max\left\{ dist\left( v,w \right):w\in C(v) \right\}}$ | $p\left( v \right)=\left\vert V(C\left( v \right)) \right\vert/\left\vert V \right\vert$, where *C(v)* denotes a component which contains vertex *v* |
| **Closeness** | $\sum_{w\in V} \frac{1}{dist(v,w)}$ |  |
| **Radiality** | $\frac{p(v)\sum_{w\in C(v)} \left( \Delta_{C(v)}+1-dist(v,w) \right)}{\left\vert V(C\left( V \right)) \right\vert-1}$ | $\Delta_{c(v)}$ denotes the maximum distance between any two vertices of the component *C(v)* |
| **Betweenness** | $\sum_{s\neq t\neq v} \frac{\sigma_{st}(v)}{\sigma_{st}}$ | $\sigma_{st}$ denotes the number of shortest paths from vertices *s* to *t* |
| **Stress** | $\sum_{s\neq t\neq v} \sigma_{st}(v)$ | $\sigma_{st}(v)$ denotes the number of shortest paths from vertices *s* to *t* which use *v* |
| **Clustering coefficient** | *C_n_* = 2*e_n_*/(*k_n_*(*k_n_*-1)) | *k_n_* is the number of neighbors of *n* and *e_n_* is the number of connected pairs between all neighbors of *n*. The clustering coefficient value of a node is a number between [0,1] |

1. Barabasi, A.L. and Z.N. Oltvai, *Network biology: understanding the cell's functional organization.* Nat Rev Genet, 2004. **5**(2): p. 101-13.

2. Watts, D.J. and S.H. Strogatz, *Collective dynamics of small-world networks.* Nature, 1998. **393**(6684): p. 440-442.

3. Junker, B. and F. Schreiber, *Analysis of Biological Networks*. 2008: Wiley-Interscience.

4. Lin, C.Y., et al., *Hubba: hub objects analyzer--a framework of interactome hubs identification for network biology.* Nucleic Acids Res, 2008. **36**(Web Server issue): p. W438-43.

**Part B**

**Constraint-based Analysis:**

Here we have described a brief definition about four constraint-based analysis performed in the paper [5-8].

| **Constraint-based analysis** | **Definition** |
| --- | --- |
| **Flux Balance Analysis (FBA)** | FBA is mathematical method for analyzing the flow of metabolites in a metabolic network. Representing a metabolic network as a stoichiometric set of equations and implying the steady state, it is possible to represent it as a stoichiometric set of equations. Since metabolic networks typically have more reactions than metabolites, this leads to an under-determined system of linear equations containing more variables than equations. Using linear programming is a standard approach to solve under-determined systems. It minimizes/maximizes an objective function as follows:  Min/Max: $\sum c_{i}.\left\vert v_{i} \right\vert$  Subject to: $S.v=0 a_{i}<v_{i}<b_{i}$  Where *c* is stoichiometric coefficient of metabolite *i* in the reaction *v*. |
| **Flux Variability Analysis (FVA)** | Biological systems often contain redundancies that contribute to their robustness. FVA could be used to examine theseredundancies by calculating the range of numerical values for every reaction flux in a network. This is carried out byoptimizing for a particular objective, while still satisfying the given constraints set on the system. |
| **Parsimonious FBA (pFBA)** | pFBAis used to label all metabolic genes based on its ability to contribute to the optimal growth rate predictions and its flux level. It classifies as follows: essential genes, pFBA optima (which includes genes that are predicted to be used for optimal growth), ELE(which includes genes that will increase cellular metabolic flux if used), MLE(which includes genes predicted to decrease the growth rate if used), and pFBA no-flux(which includes genes that cannot be used in the given growth conditions). |
| **Single gene deletion** | Gene deletion effect on cellular growth could be simulated similar to linear optimization ofgrowth. The upper and lower flux bounds for the reaction(s) corresponding to the deleted gene are both set to zero. In the case of association of a singlegene with multiple reactions, the gene deletion should cause removal of all associated reactions.In addition, a reaction that could be catalyzed by multiple gene products will not be removed in a single genedeletion. |

1. Barabasi, A.L. and Z.N. Oltvai, *Network biology: understanding the cell's functional organization.* Nat Rev Genet, 2004. **5**(2): p. 101-13.

2. Watts, D.J. and S.H. Strogatz, *Collective dynamics of small-world networks.* Nature, 1998. **393**(6684): p. 440-442.

3. Junker, B. and F. Schreiber, *Analysis of Biological Networks*. 2008: Wiley-Interscience.

4. Lin, C.Y., et al., *Hubba: hub objects analyzer--a framework of interactome hubs identification for network biology.* Nucleic Acids Res, 2008. **36**(Web Server issue): p. W438-43.

5. Mahadevan, R. and C.H. Schilling, *The effects of alternate optimal solutions in constraint-based genome-scale metabolic models.* Metabolic Engineering, 2003. **5**(4): p. 264-276.

6. Orth, J., I. Thiele, and B. Palsson, *What is flux balance analysis?* Nature Biotechnology, 2010. **28**(3): p. 245-248.

7. Becker, S., et al., *Quantitative prediction of cellular metabolism with constraint-based models: the COBRA Toolbox.* Nat. Protocols, 2007. **2**(3): p. 727-738.

8. Schellenberger, J., et al., *Quantitative prediction of cellular metabolism with constraint-based models: the COBRA Toolbox v2.0.* Nat Protoc, 2011. **6**(9): p. 1290-307.
