# Supplementary material for: Computational Analysis of Reciprocal Association of Metabolism and Epigenetics in the Budding Yeast: A Genome-Scale Metabolic Model (GSMM) Approach
Source: PLoS One. 2014 Nov 3;9(11):e111686. doi: 10.1371/journal.pone.0111686 (PMC4218804; doi:10.1371/journal.pone.0111686)
Supplement: File S3 — Up- and down regulated genes and their corresponding reactions according to GPR in nine mutated histone tail models. (DOCX) [file pone.0111686.s003.docx]

Down regulated genes related to group 1

| Gene name | Reaction in (GPR) | Function | Fold Change |
| --- | --- | --- | --- |
| Q0130 | r_0226 | ATP synthase | -2.452231089 |
| YNL268W | r_1213 | L-lysine transport | -1.203603268 |
| YEL063C | r_1184  r_1238 | L-arganine transport  orntithine transport | -1.168367068 |
| YML008C | r_0986 | S-adenosyl-methionine delta-24-sterol-c-methyltransferase | -0.654855569 |
| YBL076C | r_0665 | isoleucyl-tRNAsynthetase | -0.485389074 |

Down regulated genes related to group 2:

| YBR006W | r_1023 | succinate-semialdehyde dehydrogenase (NADP) | -3.69014 |
| --- | --- | --- | --- |
| YJL167W | r_0355  r_0462 | Dimethylallyltranstransferase  geranyltranstransferase | -2.21536 |
| YJR016C | r_0352  r_0353 | dihydroxy-acid dehydratase  dihydroxy-acid dehydratase | -1.88152 |
| YHR128W | r_1074 | uracil phosphoribosyltransferase | -1.52671 |
| YBR084W | r_0447  r_0724  r_0733 | formate-tetrahydrofolate ligase  methenyltetrahydrifikatecyclohydrolase  methylenetetrahydrofolate dehydrogenase | -1.36244 |
| YOL068C | r_0767 | NAD nucleosidase nuclear | -1.35882 |
| YHR216W | r_0565 | IMP dehydrogenase | -1.31856 |
| YEL046C | r_0675  r_1040 | L-allo-threonine aldolase  threonine aldolase | -1.21709 |
| YBR256C | r_0968 | riboflavin synthase | -1.19558 |
| YOR347C | r_0962 | pyruvate kinase | -1.19496 |
| YHR096C | r_1134  r_1139  r_1166 | D-fructose transport  D-mannose transport  glucose transport | -1.17628 |
| YJL045W | r_1021 | succinate dehydrogenase (ubiquinone-6) | -1.11557 |
| YML075C | r_0558 | hydroxymethylglutaryl CoA reductase | -1.08612 |
| YIL010W | r_1037 | thioredoxin | -1.05396 |
| YGR209C | r_0550  r_0552  r_0883  r_0970  r_0971  r_0972  r_0973  r_1037  r_1038 | hydrogen peroxide reductase  hydrogen peroxide reductase  phosphoadenylyl-sulfate reductase  ribonucleoside-triphosphate reductase (ATP)  ribonucleoside-triphosphate reductase (CTP)  ribonucleoside-triphosphate reductase (GTP)  ribonucleoside-triphosphate reductase (UTP)  thioredoxin  thioredoxinreductase | -1.04681 |
| YML022W | r_0139 | adenine phosphoribosyltransferase | -1.0427 |
| YDR513W | r_0481  r_0483 | glutathione oxidoreductase  glutathione peridoxase | -1.03308 |
| YPR006C | r_0734 | methylisocitratelyase | -1.01581 |
| YIL145C | r_0843 | pantothenate synthase | -1.01536 |
| YPL104W | r_0221 | Aspartyl-tRNAsynthetase | -0.95751 |
| YDR305C | r_0204 | Ap4A hydrolase | -0.9528 |
| YPL231W | r_0098  r_0385  r_0386  r_0387  r_0389  r_0391  r_0395  r_0397  r_0398  r_0415  r_0432  r_0433  r_0434  r_0435  r_0720 | acetyl-CoA ACP transacylase  fatty acid synthase (n-C10:0)  fatty acid synthase (n-C12:0)  fatty acid synthase (n-C14:0)  fatty acid synthase (n-C16:0)  fatty acid synthase (n-C18:0)  fatty acid synthase (n-C8:0)  fatty acyl-CoA synthase (n-C10:0CoA)  fatty acyl-CoA synthase (n-C8:0CoA)  fatty-acyl-ACP hydrolase  fatty-acyl-CoA synthase (n-C12:0CoA)  fatty-acyl-CoA synthase (n-C14:0CoA)  fatty-acyl-CoA synthase (n-C16:0CoA)  fatty-acyl-CoA synthase (n-C18:0CoA)  malonyl-CoA-ACP transacylase | -0.94576 |
| YHR072W | r_0698 | lanosterol synthase | -0.93331 |
| YDR019C | r_0501  r_1030 | glycine cleavage system  tetrahydrofolateaminomethyltransferase | -0.90234 |
| YMR101C | r_0281 to r_0299 | cis-prenyltransferase step 01 to step 19 | -0.84104 |
| YEL038W | r_0013 | 2,3-diketo-5-methylthio-1-phosphopentane degradation reaction | -0.84045 |
| YOR143C | r_1034  r_1035 | thiamine diphosphokinase  thiamine-diphosphate kinase | -0.81237 |
| YMR217W | r_0514 | GMP synthase | -0.80001 |
| YLR133W | r_0273  r_0368 | choline kinase  ethanolamine kinase | -0.72271 |
| YOR273C | r_1251  r_1260  r_1263 | putrescine transport  spermidine transport  spermine transport | -0.69498 |
| YBR117C | r_1049  r_1050 | transketolase 1  transketolase 2 | -0.66659 |
| YMR113W | r_0346 | dihydrofolate synthase | -0.6456 |
| YGL253W | r_0533  r_0534  r_0535 | hexokinase (D-fructose:ATP)  hexokinase (D-glucose:ATP)  hexokinase (D-mannose:ATP) | -0.63732 |
| YKL194C | r_1043 | threonyl-tRNAsynthetase | -0.61347 |
| YER015W | r_0399  r_0400  r_0402  r_0404  r_0410  r_0412  r_0414 | fatty-acid--CoA ligase (decanoate)  fatty-acid--CoA ligase (dodecanoate)  fatty-acid--CoA ligase (hexadecanoate)  fatty-acid--CoA ligase (hexadecanoate)  fatty-acid--CoA ligase (octanoate)  fatty-acid--CoA ligase (tetradecanoate)  fatty-acid--CoA ligase (tetradecanoate) | -0.56708 |
| YKL174C | r_1250  r_1259 | putrescine excretion  spermidine excretion | -0.50741 |
| YML008C | r_0986 | S-adenosyl-methionine delta-24-sterol-c-methyltransferase | -0.48062 |
| YGL026C | r_1055 | tryptophan synthase | -0.46411 |

Down regulated genes related to group 3:

| YER005W | r_0227  r_0788  r_0804 | ATPase, cytosolic  nucleoside diphosphatase  nucleoside triphosphatase | -1.42287 |
| --- | --- | --- | --- |
| YOR143C | r_1034  r_1035 | thiamine diphosphokinase  thiamine-diphosphate kinase | -1.30696 |
| YPL061W | r_0173  r_0177 | aldehyde dehydrogenase(3-aminopropanal, NAD)  aldehyde dehydrogenase (indole-3-acetaldehyde, NADP) | -1.23544 |
| YML022W | r_0139 | adenine phosphoribosyltransferase | -1.22283 |
| YDR454C | r_0330  r_0528  r_0529 | deoxyguanylate kinase (dGMP:ATP)  guanylate kinase  guanylate kinase (GMP:dATP) | -1.1885 |
| YNL256W | r_0017  r_0347  r_0350 | 2-amino-4-hydroxy-6-hydroxymethyldihydropteridinediphosphokinase  dihydroneopterinaldolase  dihydropteroate synthase | -1.09926 |
| YMR246W | r_0401  r_0407  r_0411 | fatty-acid--CoA ligase (hexadecanoate)  fatty-acid--CoA ligase (octadecanoate)  fatty-acid--CoA ligase (tetradecanoate) | -1.07511 |
| YOL068C | r_0767 | NAD nucleosidase nuclear | -0.96247 |
| YGR264C | r_0729 | methionyl-tRNAsynthetase | -0.9399 |
| YDR305C | r_0204 | Ap4A hydrolase | -0.91434 |
| YLL048C | r_1028 | taurcholate via ABC system | -0.89156 |
| YPR062W | r_0318 | cytosine deaminase | -0.78461 |
| YBR084W | r_0447  r_0724  r_0733 | formate-tetrahydrofolate ligase  methenyltetrahydrifikatecyclohydrolase  methylenetetrahydrofolate dehydrogenase (NADP) | -0.74675 |
| YOR175C | r_0008 | 1-acyl-sn-gylcerol-3-phosphate acyltransferase | -0.66897 |
| YMR217W | r_0514 | GMP synthase | -0.57915 |
| YGR175C | r_1010  r_1011 | squaleneepoxidase (NAD)  squaleneepoxidase (NADP) | -0.54403 |
| YMR145C | r_0770 | NADH dehydrogenase, cytosolic/mitochondrial | -0.29841 |

Down regulated genes related to group 4:

| YPL061W | r_0173  r_0177 | aldehyde dehydrogenase (acetaldehyde, NADP)  aldehyde dehydrogenase (indole-3-acetaldehyde, NADP) | -2.11104 |
| --- | --- | --- | --- |
| YHR144C | r_0326  r_0327 | dCMPdeaminase  dCTPdeaminase | -1.47374 |
| YOL061W | r_0916 | phosphoribosylpyrophosphatesynthetase | -1.35119 |
| YBR293W | r_1185  r_1200  r_1212  r_1221 | L-arganine transport  L-histidine transport, vacuolar  L-lysine transport  L-tyrosine transport | -1.27739 |
| YML075C | r_0558 | hydroxymethylglutaryl CoA reductase | -1.21076 |
| YNR041C | r_0555 | hydroxybenzoateoctaprenyltransferase | -1.13744 |
| YNL292W | r_1095 | yUMPsynthetase | -1.11252 |
| YDR380W | r_0854 | phenylpyruvate decarboxylase | -1.02728 |
| YPL078C | r_0226 | ATP synthase | -1.02069 |
| YDL090C | r_0249 | CAAX farnesyltransferase | -1.00879 |
| YMR162C | r_1143 | DNF1 flippase | -0.8988 |
| YDR377W | r_0226 | ATP synthase | -0.89655 |
| YMR202W | r_0243 | C-8 sterol isomerase | -0.82515 |
| YKR043C | r_2126 | sedoheptulosebisphosphatase | -0.78687 |
| YOL068C | r_0767 | NAD nucleosidase nuclear | -0.59649 |
| YDR376W | r_0530 | Heme O monooxygenase | -0.50654 |
| YFL055W | r_1190  r_1196  r_1217 | L-aspartate transport  L-glutamate transport  L-serine transport | -0.19711 |

Down regulated genes related to group 5:

| YMR113W | r_0346 | dihydrofolate synthase | -0.73078 |
| --- | --- | --- | --- |
| YCR037C | r_1244 | phosphate transport | -0.62838 |
| YMR041C | r_0320 | D-arabinose 1-dehydrogenase (NAD) | -0.60558 |
| YJL121C | r_0984 | ribulose 5-phosphate 3-epimerase | -0.47676 |
| YFR033C | r_0439 | ferrocytochrome-c:oxygenoxidoreductase | -0.38815 |

Down regulated genes related to group 6:

| YJR025C | r_0058 | 3-hydroxyanthranilate 3,4-dioxygenase | -1.50175 |
| --- | --- | --- | --- |
| YOR071C | r_1270 | thiamine transport | -1.25917 |
| YJL045W | r_1021 | succinate dehydrogenase (ubiquinone-6) | -1.03114 |
| YNL202W | r_0375  r_0377  r_0379  r_0380  r_0382 | fatty acid oxidation  fatty acid oxidation  fatty acid oxidation  fatty acid oxidation  fatty acid oxidation | -0.87093 |
| YNL142W | r_1115 | ammonia transport | -0.74132 |
| YER015W | r_0399  r_0400  r_0402  r_0404  r_0410  r_0412  r_0414 | fatty-acid--CoA ligase (decanoate)  fatty-acid--CoA ligase (dodecanoate)  fatty-acid--CoA ligase (hexadecenoate)  fatty-acid--CoA ligase (hexadecenoate)  fatty-acid--CoA ligase (octanoate)  fatty-acid--CoA ligase (tetradecanoate)  fatty-acid--CoA ligase (tetradecenoate) | -0.69312 |
| YML054C | r_0004 | (S)-lactate:ferricytochrome-c 2-oxidoreductase | -0.67901 |
| YBL098W | r_0671 | kynurenine 3-monooxygenase | -0.60716 |
| YAL022C | r_1109  r_1132 | adenosine transport  cytidine transport | -0.43519 |

Down regulated genes related to group 7:

| Q0130 | r_0226 | ATP synthase | -4.16844 |
| --- | --- | --- | --- |
| YMR011W | r_1134  r_1139  r_1166 | D-fructose transport  D-mannose transport  glucose transport | -1.19668 |
| YBR093C | r_0116 | acid phosphatase (secreted) | -1.05613 |
| YCR098C | r_1169  r_1170 | glycero-3-phospho-1-inositol transport  glycero-3-phosphocholine transport | -0.8102 |
| YGR264C | r_0729 | methionyl-tRNAsynthetase | -0.71561 |
| YDR380W | r_0854 | phenylpyruvate decarboxylase | -0.69368 |
| YBR002C | r_0281  r_0282  r_0283  r_0284  r_0285  r_0286  r_0287  r_0288  r_0289  r_0290  r_0291  r_0292  r_0293  r_0294  r_0295  r_0296  r_0297  r_0298  r_0299 | cis-prenyltransferase step 01  cis-prenyltransferase step 02  cis-prenyltransferase step 03  cis-prenyltransferase step 04  cis-prenyltransferase step 05  cis-prenyltransferase step 06  cis-prenyltransferase step 07  cis-prenyltransferase step 08  cis-prenyltransferase step 09  cis-prenyltransferase step 10  cis-prenyltransferase step 11  cis-prenyltransferase step 12  cis-prenyltransferase step 13  cis-prenyltransferase step 14  cis-prenyltransferase step 15  cis-prenyltransferase step 16  cis-prenyltransferase step 17  cis-prenyltransferase step 18  cis-prenyltransferase step 19 | -0.65492 |
| YGR157W | r_0859 | phosphatidylethanolaminemethyltransferase | -0.63773 |
| YBR069C | r_1192  r_1201  r_1205  r_1211  r_1218  r_1219  r_1223  r_1224 | L-cystine transport  L-histidine transport  L-isoleucine transport  L-leucine transport  L-threonine transport  L-tryptophan transport  L-tyrosine transport  L-valine transport | -0.60329 |
| YBR068C | r_1183  r_1192  r_1205  r_1211  r_1214  r_1215  r_1219  r_1223  r_1224 | L-alanine transport  L-cystine transport  L-isoleucine transport  L-leucine transport  L-methionine transport  L-phenylalanine transport  L-tryptophan transport  L-tyrosine transport  L-valine transport | -0.58262 |
| YNR012W | r_0315  r_0077  r_0078 | cytidine kinase (GTP)  uridine kinase (ATP:uridine)  uridine kinase (GTP:uridine) | -0.56279 |
| YDL095W | r_0362 | dolichyl-phosphate-mannose-protein mannosyltransferase | -0.55177 |
| YKR072C | r_0906 | phosphopantothenoylcysteine decarboxylase | -0.51929 |
| YML106W | r_0820 | orotatephosphoribosyltransferase | -0.41685 |
| YFL045C | r_0902 | phosphomannomutase | -0.39685 |
| YDR023W | r_0995 | seryl-tRNAsynthetase | -0.39058 |
| YLR372W | r_0393  r_0394 | fatty acid synthase (n-C24:0)  fatty acid synthase (n-C26:0) | -0.35492 |
| YDL004W | r_0226 | ATP synthase | -0.34385 |
| YHL011C | r_0916 | phosphoribosylpyrophosphatesynthetase | -0.33296 |
| YOR335C | r_0157 | alanyl-tRNAsynthetase | -0.30981 |
| YGR208W | r_0917 | phosphoserine phosphatase (L-serine) | -0.29663 |
| YOR168W | r_0478 | glutaminyl-tRNAsynthetase | -0.27732 |
| YDR226W | r_0148 | adenylate kinase | -0.27352 |
| YPL078C | r_0226 | ATP synthase | -0.26243 |

Down regulated genes related to group 8:

| Q0130 | r_0226 | ATP synthase | -2.97345 |
| --- | --- | --- | --- |
| YPL212C | r_1095 | yUMPsynthetase | -0.17054 |

Down regulated genes related to group 9:

| YGR264C | r_0729 | methionyl-tRNAsynthetase | -3.10398 |
| --- | --- | --- | --- |
| YLR372W | r_0393  r_0394 | fatty acid synthase (n-C24:0)  fatty acid synthase (n-C26:0) | -2.70432 |
| YEL017C-A | r_0227 | ATPase, cytosolic | -2.68801 |
| YDL095W | r_0362 | dolichyl-phosphate-mannose-protein mannosyltransferase | -2.24316 |
| YGL148W | r_0279 | chorismate synthase | -2.13659 |
